# A functional variant in *NEPH3* gene confers high risk of renal failure in primary hematuric glomerulopathies. Evidence for predisposition to microalbuminuria in the general population

**DOI:** 10.1371/journal.pone.0174274

**Published:** 2017-03-23

**Authors:** Konstantinos Voskarides, Charalambos Stefanou, Myrtani Pieri, Panayiota Demosthenous, Kyriakos Felekkis, Maria Arsali, Yiannis Athanasiou, Dimitris Xydakis, Kostas Stylianou, Eugenios Daphnis, Giorgos Goulielmos, Petros Loizou, Judith Savige, Martin Höhne, Linus A. Völker, Thomas Benzing, Patrick H. Maxwell, Daniel P. Gale, Mathias Gorski, Carsten Böger, Barbara Kollerits, Florian Kronenberg, Bernhard Paulweber, Michalis Zavros, Alkis Pierides, Constantinos Deltas

**Affiliations:** 1 Department of Biological Sciences and Molecular Medicine Research Center, University of Cyprus, Nicosia, Cyprus; 2 Department of Nephrology, Limassol General Hospital, Limassol, Cyprus; 3 Department of Nephrology, Nicosia General Hospital, Nicosia, Cyprus; 4 Department of Nephrology, University of Crete, Heraklion, Crete, Greece; 5 Department of Internal Medicine, Section of Molecular Medicine and Human Genetics, Medical School, University of Crete, Heraklion, Greece; 6 Private Clinical Laboratory, Paralimni, Cyprus; 7 Department of Medicine, The University of Melbourne, Northern Health, Epping, Australia; 8 Department 2 of Internal Medicine and Center for Molecular Medicine Cologne, University of Cologne, Cologne, Germany; 9 Systems Biology of Ageing Cologne (Sybacol), University of Cologne, Cologne, Germany; 10 Cologne Excellence Cluster on Cellular Stress Responses in Aging-Associated Diseases, University of Cologne, Cologne, Germany; 11 Division of Medicine, University College London, London, United Kingdom; 12 UCL Centre for Nephrology, University College London, London, United Kingdom; 13 Department of Nephrology, University Hospital Regensburg, Regensburg, Germany; 14 Department of Genetic Epidemiology, University Regensburg, Regensburg, Germany; 15 Division of Genetic Epidemiology, Department of Medical Genetics, Molecular and Clinical Pharmacology, Innsbruck Medical University, Innsbruck, Austria; 16 First Department of Internal Medicine, Paracelsus Private Medical University Salzburg, Salzburg, Austria; 17 Department of Nephrology, Hippocrateon Hospital, Nicosia, Cyprus; "INSERM", FRANCE

## Abstract

**Background:**

Recent data emphasize that thin basement membrane nephropathy (TBMN) should not be viewed as a form of benign familial hematuria since chronic renal failure (CRF) and even end-stage renal disease (ESRD), is a possible development for a subset of patients on long-term follow-up, through the onset of focal and segmental glomerulosclerosis (FSGS). We hypothesize that genetic modifiers may explain this variability of symptoms.

**Methods:**

We looked *in silico* for potentially deleterious functional SNPs, using very strict criteria, in all the genes significantly expressed in the slit diaphragm (SD). Two variants were genotyped in a cohort of well-studied adult TBMN patients from 19 Greek-Cypriot families, with a homogeneous genetic background. Patients were categorized as “Severe” or “Mild”, based on the presence or not of proteinuria, CRF and ESRD. A larger pooled cohort (HEMATURIA) of 524 patients, including IgA nephropathy patients, was used for verification. Additionally, three large general population cohorts [Framingham Heart Study (FHS), KORAF4 and SAPHIR] were used to investigate if the *NEPH3*-V353M variant has any renal effect in the general population.

**Results and conclusions:**

Genotyping for two high-scored variants in 103 TBMN adult patients with founder mutations who were classified as mildly or severely affected, pointed to an association with variant *NEPH3*-V353M (filtrin). This promising result prompted testing in the larger pooled cohort (HEMATURIA), indicating an association of the 353M variant with disease severity under the dominant model (p = 3.0x10^-3^, OR = 6.64 adjusting for gender/age; allelic association: p = 4.2x10^-3^ adjusting for patients’ kinships). Subsequently, genotyping 6,531 subjects of the Framingham Heart Study (FHS) revealed an association of the homozygous 353M/M genotype with microalbuminuria (p = 1.0x10^-3^). Two further general population cohorts, KORAF4 and SAPHIR confirmed the association, and a meta-analysis of all three cohorts (11,258 individuals) was highly significant (p = 1.3x10^-5^, OR = 7.46). Functional studies showed that Neph3 homodimerization and Neph3-Nephrin heterodimerization are disturbed by variant 353M. Additionally, 353M was associated with differential activation of the unfolded protein response pathway, when overexpressed in stressed cultured undifferentiated podocyte cells, thus attesting to its functional significance. Genetics and functional studies support a “rare variant-strong effect” role for *NEPH3*-V353M, by exerting a negative modifier effect on primary glomerular hematuria. Additionally, genetics studies provide evidence for a role in predisposing homozygous subjects of the general population to micro-albuminuria.

## Introduction

Podocytes are terminally differentiated epithelial cells with multiple foot processes. A highly specialized cell junction, known as the slit diaphragm (SD), links adjacent foot processes of podocytes and is considered to be the most important selective barrier to protein leakage into the glomerular filtrate[1, 2].

Nephrin and Nephrin-like proteins (Neph) are considered as the most significant parts of the SD. Nephrin-like proteins comprise a family of transmembranous proteins that belong to the immunoglobulin superfamily[3–5] due to the Ig-like domains through which they promote protein-protein interactions. In podocytes, Neph3 (filtrin), like the other Neph proteins and nephrin, localizes at the SD[6–10]. Nephrin and nephrin-neph complexes appear to be key components of the SD since: a) nephrin deficiency results in the absence of SD and in massive proteinuria in humans and mice, causing the Finish type nephrotic syndrome[11–13], b) dissociation of nephrin-neph1 complex by antibodies results in proteinuria in mice[14]. Similarly, in Neph1-deficient mice, the podocyte foot processes are effaced and the mice exhibit severe proteinuria[15]. It has been shown that Neph3 forms homodimers and heterodimers with the proteins podocin, ZO1, Nephrin and Neph1. Interaction of Neph3 with Neph1 induces cell adhesion[3, 16–18]. In addition, similarly to nephrin mRNA, the expression of Neph3 is down-regulated in human proteinuric diseases, suggesting a probable role in maintaining normal SD structure and function[10].

We hypothesize that the phenotypic heterogeneity seen in primary hematurias of glomerular origin is partly explained by modifier genes, related with the SD structure[19–21]. Microscopic hematuria, sporadic or familial, is a frequent condition but with underestimated risks[21–30]. Recent findings by us and others showed that about 50% of patients with *COL4A3/COL4A4* heterozygous mutations, a prevalent cause of familial microscopic hematuria due to TBMN, develop proteinuria with secondary focal segmental glomerulosclerosis (FSGS), after their third decade of life. In a significant percentage of patients this is followed by chronic or end-stage renal disease (CRF/ESRD) mostly at ages over 50-years[19, 27, 29–37] (other genetic causes of familial hematuria that have been identified include mutations in the *CFHR5*[38, 39]and *MYH9*[40] genes). Approximately half of the patients have a benign outcome with lifetime microscopic hematuria or microscopic hematuria plus minimal proteinuria and normal kidney function. Conversely, the majority of non-heritable glomerular hematuria cases are attributable to IgA nephropathy. About 9–50% of IgA nephropathy patients progress to ESRD within 20 years of onset[41, 42], [43]. In TBMN and Alport syndrome, studies in animal models support the existence of genetic loci that influence disease progression[44, 45]. In humans, we and others have reported the association of the *NPHS2*-R229Q variant (in podocin, a SD component) with proteinuria and CRF in patients with TBMN[46] and familial hematuria[47]. Modifier genes have been identified in other human inherited renal diseases, such as polycystic kidney disease and renal ciliopathies[48, 49]. Despite this, other environmental or co-morbidity factors may contribute to the progression in familial hematuria patients, including nutrition (e.g. high fat diet), hypertension, obesity, diabetes etc. None of these factors have been studied in depth yet.

We took advantage of founder phenomena we observed in Cyprus in order to see if genetic variants on important slit-diaphragm genes can serve as modifiers of disease severity in a cohort of 103 adult TBMN patients, who are heterozygous for known *COL4A3* or *COL4A4* mutations. A rigorous *in silico* analysis followed by genetic testing of several candidates on ten genes, resulted in an evolutionarily conserved single variant with indicative statistical significance, in the *NEPH3* gene, namely p.V353M. The role of this same variant was further supported in independent cohorts of primary hematuria while it also emerged as a likely DNA variant that may be predisposing subjects of the general population to microalbuminuria, when in homozygosity. Functional experiments in cultured cells enhanced the suggested significance of this genetic variant, which apparently acts as a hypomorphic mutation. This is the first study that genetically links Neph proteins with human renal disease.

## Subjects and methods

### Study cohorts

HEMATURIA is a pooled hematuric cohort that includes four sub-cohorts (A, B, C and D). Sub-cohort A comprised 103 patients with TBMN from 19 large Greek-Cypriot families[27] (and unpublished data) and was used for an initial screening of the candidate genetic variants, in order to decide which SNP/s would be genotyped in additional samples. Importantly, in this sub-cohort A, 78 patients are heterozygous for a common founder *COL4A3*-p.G1334E mutation, 19 of 103 are heterozygous for the *COL4A3*-p.G871C mutation and 6 of 103 are heterozygous for the *COL4A4*-c.3854delG mutation. Since young individuals with apparently mild disease could develop severe disease at older age, patients with “mild disease” (see below) and younger than 50-yo (born after January 1963) were excluded. The additional three hematuric sub-cohorts were: Sub-cohort B: 69 familial microscopic hematuria cases, initially of unknown etiology, belonging to 37 families. In some of these patients a *COL4A3* or a *COL4A4* mutation has been found after the start of this project, but none of these mutations was a founder. Sub-cohort C: 34 unrelated familial or sporadic microscopic hematuria cases (with a TBMN biopsy or with genetic studies in which hematuria segregated in their family with the *COL4A3/COL4A4* locus or mutations, or both) provided by Prof. Judy Savige (Australia). Sub-cohort D: 318 unrelated patients with biopsy-proven IgA nephropathy from Crete-Greece (72 patients) and UK (246 patients). Information for all sub-cohorts can be found in Table 1. Testing of DNA from 462 healthy anonymous individuals from our DNA Biobank (174 from UK and the rest from Cyprus), showed that the *NEPH3*-353M allele frequency was 2.9% among UK and 2.6% among Cypriots. The study was designed and performed according to provisions of Declaration of Helsinki. The study was approved by the Cyprus National Bioethics Committee and participants gave their signed informed consent.

**Table 1:** Characteristics of the pooled HEMATURIA cohort.

Cohort	Origin	N	Mild				Severe			
			N (%)	Age: mean (SD)	Females: N (%)	With ESRD: N (%)	N (%)	Age: mean (SD)	Females: N (%)	With ESRD: N (%)
**A.** Heterozygous for collagen IV mutations^a^	Cyprus	103	44 (43%)	60.3 (±10.3)	26 (59%)	0	59 (57%)	62.6 (±12.9)	26 (44%)	20 (34%)
**B.** Familial cases of MH^b^	Cyprus, Greece	69	35 (50%)	53.7 (±8.7)	28 (80%)	0	34 (50%)	55.8 (±12.5)	13 (38%)	12 (35%)
**C.** Familial or sporadic cases of MH^c^	Australia	34	22 (65%)	53.8 (±9.7)	15 (41%)	0	12 (35%)	44.9 (±14.8)	9 (75%)	0
**D.** Patients with IgA nephropathy	Crete (Greece)UK	318	122 (38%)	40.2 (±11.1)	51 (42%)	0	196 (62%)	44.4 (±12.3)	54 (28%)	66 (34%)

MH: Microscopic Hematuria, ESRD: End-Stage Renal Disease

^a^ Of 103 carriers, 78 carried mutation *COL4A3*-p.G1334E, 19 carried mutation *COL4A3*-p.G871C and six carried mutation *COL4A4*-c.3854delG

^b^ “Mild” patients born before 01/1963

^c^ “Mild” patients born before 01/1968. For A, B, C sub-cohorts, age mean difference for Mild and Severe is not significant (p = 0.298).

Furthermore, we investigated the possible renal effect of *NEPH3*-p.V353M in the general population using three independent population-based samples: 6,351 DNA samples from the Framingham Heart Study, 3,037 samples from the German KORAF4 and 1,690 samples from Austrian SAPHIRstudy[50–52]. For a detailed description of these studies, see Text A in S1 File. Our main investigation in those cohorts was for microalbuminuria, a sensitive biomarker for renal damage. All participants in these cohorts gave their signed informed consent.

### Clinical assessment and study outcomes

Patients of HEMATURIA cohort were classified as “Mild” or “Severe”. For sub-cohorts A, B, C, “Mild” patients had only microscopic or macroscopic hematuria episodes but no chronic renal failure, or hematuria plus low grade proteinuria (<300 mg/24 hrs, but no chronic renal failure). An age limit was determined for this category as explained above (see also Table 1), thereby excluding 25, 42 and 126 young patients from initial sub-cohorts A, B and C. For A, B, C sub-cohorts, patients with severe disease had hematuria plus proteinuria ≥500 mg/24 hrs or hematuria plus proteinuria plus chronic renal failure or ESRD. Renal failure was defined as an elevated serum creatinine over 1.5 mg/dl. Patients with borderline proteinuria and another concomitant renal disease (*e*.*g*. over five years of diabetes, vesicoureteric reflux etc), or at the extreme of body weight (outside ±2 SD of the cohort mean) were excluded. For sub-cohort D (IgAN) information of proteinuria was not available for most of the patients, so we classified as “Severe” patients with chronic renal impairment (eGFR < 45 mL/min, calculated by the MDRD formula) or ESRD and as “Mild” patients with eGFR ≥ 45 mL/min, (calculated by the MDRD formula) at least 5 years after diagnosis. Patients not falling into these categories (i.e. those in whom renal function was not known to be unaffected at least 5 years after diagnosis) were excluded. UK patients were from the MRC/Kidney Research UK National DNA Bank for Glomerulonephritis. The final numbers of patients that included in the genetic analysis (after excluding the patients that did not fulfill the above criteria) can be seen in Tables 1 and 2.

**Table 2:** Frequencies and statistical analysis of variant *NEPH3*-p.V353M in the various hematuric sub-cohorts, based on disease severity.

		Genotype counts			Genotype frequency			Allele counts		Allele frequency		Statistics		
Cohort	N	VV	VM	MM	VV	VM	MM	V	M	V	M	“Mild” v. “Severe”^a^	“Mild” v. “Severe”^b^	Odds Ratio (Dominant model)
General population	462	437	25	0	0.946	0.054	0	899	25	0.973	0.027			
ExAC Browser	54373	52712	1595	61	0.969	0.029	0.002	105434	3312	0.969	0.031			
**Mild**														
A	44	44	0	0	1.0	0	0	88	0	1.0	0			
B	35	35	0	0	1.0	0	0	70	0	1.0	0			
C	22	22	0	0	1.0	0	0	44	0	1.0	0			
D	122	119	3	0	0.975	0.025	0	241	3	0.988	0.012			
**Total**	**223**	**220**	**3**	**0**	0.987	0.013	0	**443**	**3**	0.993	0.007			
**Severe**														
A	59	53	6	0	0.898	0.102	0	112	6	0.949	0.051	^c^**3.6x10**^**-2**^		
B	34	32	2	0	0.941	0.059	0	66	2	0.971	0.029			
C	12	10	2	0	0.833	0.167	0	22	2	0.917	0.083			
D	196	181	14	1	0.923	0.071	0.006	376	16	0.959	0.041			
**Total**	**301**	**276**	**24**	**1**	0.917	0.080	0.003	**576**	**26**	0.957	0.043	^c^**3.0x10**^**-4**^	^c^**2.0x10**^**-4**^	**6.64** (1.98, 22.29)
												^d^**3.0x10**^**-3**^	^e^**4.2x10**^**-3**^	^d^**6.63** (1.94, 22.68)

The four sub-cohorts presented here comprise the larger HEMATURIA cohort. Description of sub-cohorts can be found in Table 1.

^a^ Genotypic association analysis p-values (dominant model);

^b^ Allelic association analysis p-values;

^c^ Fisher’s Exact Test (2-sided);

^d^ Adjusted for gender and age;

^e^ Adjusted for patients’ kinships

For FHS sample group (cohort 2), immuno-turbimetry was used for measuring urine albumin concentration (Tina-quant Albumin assay; Roche Diagnostics, Indianapolis, IN). Urinary creatinine concentration was measured with the modified Jaffe method; urinary albumin was indexed to urinary creatinine to account for differences in urine concentrations (UACR; g:mg/g). UACR is a reliable measure of urinary albumin excretion and correlates with albumin excretion rates obtained from 24-hour urine collection [53, 54]. Microalbuminuria was defined as a UACR of >25 mg/g for women and UACR of >17 mg/g for men[55]. The same approach was followed for cohorts KORAF4 and SAPHIR.

### Candidate genes and SNPs selection

We used Polyphen and SIFT prediction algorithms as they are found in Ensembl database (www.ensembl.org) and SNPs3D algorithm (http://www.snps3d.org/) to assess the effect of all registered non-synonymous SNPs (www.ensembl.org) located in the 10 most significant genes for the SD structure: *NPHS1*, *NPHS2*, *NEPH1*, *NEPH2*, *NEPH3*, *CD2AP*, *TRPC6*, *TJP1 (ZO1)*, *FAT1*, *FAT2*. Significance was determined based on available studies since 2011 (genetic studies, animal model studies, functional studies) that show contribution of SD-located proteins to the SD function. For example, the gene of P-cadherin, an SD-located protein, was not included in our investigation since studies have shown that it does not seem to be essential for the filtration barrier. Additionally, we had special interest to investigate the four nephrin-like proteins of the SD, since their extra-cellular Ig-like domains are considered to be the main scaffold of the SD sieve[1, 2]. We recognize that additional genes may prove to be of similar or even higher significance in the future. High scored SNPs (predicted to be “deleterious” by all three algorithms: SIFT value <0.05, Polyphen value >0.9, SNPs3D <-1.0) were searched in Ensembl database for available population genetics data. Only SNPs with global MAF ≥ 2.5% were processed for genotyping in sub-cohort A. SNPs with p-values <0.05 for sub-cohort A were genotyped in the entire pooled HEMATURIA cohort for further evaluation.

### DNA genotyping

Selected SNPs were genotyped by standard PCR-RFLP analysis (restriction enzymes from New England Biolabs, USA). PCR amplified products were digested and electrophoresed on 2–3% (depending on cleavage sizes) routine agarose gels (Table A in S1 File). The UK IgA DNA samples and the FHS DNA samples were genotyped with the KASPar method by outsourcing to KBioscience (Herts, UK). The KORAF4 and SAPHIR DNA samples were genotyped with the TaqMan assay (probes and primers available on request) in a RealTime analyzer. DNA from 12 patients was directly re-sequenced after amplifying all the 15 exons (primers available on request) of *NEPH3* gene, in an ABI PRISM^™^ 3130*xl* (California, USA) genetic analyzer.

### Statistical analysis

Genotypic and allelic statistical analysis, odds ratios and independent t-test (for age means’ comparison in Table 3) calculations were performed by SPSS v.13. P values were calculated by Pearson Chi-Square test or by Fisher’s Exact Test (2-sided) where genotype values less than 10 existed (cases: “severe” group, control: “mild” group). Logistic regression was used for adjusting for gender and age. R statistical package v.3.0.1 was used for KORAF4 and SAPHIR cohorts and for the meta-analysis. Allelic association analysis for HEMATURIA cohort was corrected for the presence of related individuals using a quasi-likelihood score function (QLS test) for estimation of allele frequencies and significance in large inbred pedigrees, based on kinship coefficients (http://www.stat.uchicago.edu/~mcpeek/software/CCQLSpackage1.3/download.html). The significance level, alpha, was set to 0.05.

**Table 3:** Demographic data of the three general population cohorts genotyped for *NEPH3*-p.V353M.

	Microalbuminuria: CASES	No Microalbuminuria: CONTROLS	P values
Cohort: **FHS**	603 (0.092)	5,928 (0.908)	
Age at urine test time	55.9 (±13.3)	46.3 (±12.2)	<0.001
Women	325 out of 603 (0.539)	3,168 out of 5,928 (0.534)	0.831
Cohort: **SAPHIR**	165 (0.098)	1,525 (0.902)	
Age at urine test time	51.5 (±5.4)	51.4(±6.0)	0.96
Women	52 out of 165 (0.315)	578 out of 1,525 (0.379)	0.11
Cohort: **KORAF4**	397 (0.131)	2,640 (0.869)	
Age at urine test time	63.8 (±13.1)	55.0 (±12.9)	< 2.2e-16
Women	160 out of 397 (0.403)	1,410 out of 2,640 (0.534)	1.01e-06

Note: Data presented as mean and SD for continuous variables and as absolute numbers and percentages for dichotomous variables. Analytical data for each of these cohorts can be found at [50–52].

### Bioinformatic analysis of neph3 (filtrin) protein and mRNA

2D protein structure prediction analysis of Neph3 protein (filtrin) and mRNA were performed by CLC Main Workbench 6 software package (www.clcbio.com), based on Hidden Markov Model algorithms. Alignments of orthologs and paralogs of the Neph3 protein were created through the same software, using ClustalW algorithm. Protein sequences were extracted from Ensembl (www.ensemble.org).

### Co-immunoprecipitation in HEK 293T cells

Cloned human *NEPH3* (filtrin) cDNA into a pCMV6 eukaryotic expression vector with available FLAG-tag sequence was purchased from Origene (Rockville, USA). *NEPH3* 353V (GTG triplet) was mutated to 353M (ATG triplet) via the Quick Change Site-Directed Mutagenesis kit (Stratagene, La Jolla, CA, USA). Constructs’ sequence was verified by DNA sequencing. Human *NPHS1* (nephrin) cDNA was cloned into a modified pcDNA6 expression vector coding for the CD5 signal peptide fused to the V5-tag sequence (sV5-tag) followed by restriction sites to insert the cDNA. Both *NEPH3* (filtrin) cDNA variants (353V, 353M) were also sub-cloned into the pcDNA6 expression vector coding for the CD5 signal peptide fused to the FLAG-tag or HA-tag sequence. Antibodies used were purchased from Sigma (FLAG), Serotec (mAb V5) and Santa-Cruz (HA). Two separate co-immunoprecipitation assays were performed: i) Neph3-Nphs1: Comparison between Flag-Neph3*-*353V binding to sV5-Nphs1 with Flag-Neph3*-*353M binding to sV5-Nphs1, ii) Neph3-Neph3 (testing homodimerization): Comparison between FLAG-Neph3*-*353V and FLAG-Neph3*-*353M when binding to HA-Neph3-353V or HA-Neph3*-*353M. The co-immunoprecipitation experimental procedure is summarized below:

HEK 293T cells were transfected using the calcium phosphate method, and incubated at 37°C for 24 h. Cells were subsequently washed with PBS, lysed in ice-cold IP-buffer (1% Triton X-100; 20 mM Tris pH 7.5; 25 mMNaCl; 50 mM NaF; 15 mM Na_4_P_2_O_7_; 1 mM EDTA; 0.25 mM PMSF; 5 mM Na_3_VO_4_) incubated on ice for 15 min and centrifuged (14.000 rpm, 4°C, 30 min). Supernatants containing equal amounts of total proteins were incubated for 1–2 h at 4°C with precipitating anti-V5 antibody (Serotec) followed by a 1–2 h incubation at 4°C with Protein G Sepharose (GE Healthcare) or with anti-FLAG M2 affinity resin (Sigma). The precipitates were washed three times with IP-buffer and bound proteins were resolved by 10% SDS-PAGE and detected by Western blot. Similar procedure was followed for anti-HA antibodies. Densitometry was performed through the publicly available ImageJ Software (http://imagej.nih.gov/ij).

### ER stress experiment: Cell culture and transfection

The AB8/13 undifferentiated podocyte cells[56], were incubated at 33°C at 5% CO_2_ and cultured in RPMI medium, supplemented with 10% Fetal Bovine Serum (FBS) (Invitrogen, California, USA), 1% of 100 units/ml Penicillin/Streptomycin (Invitrogen, CA) and 1% Insulin-Transferrin-Selenium (Invitrogen, CA). At 70% confluence, cells were transiently transfected with vectors containing the collagen cDNAs, wild type or mutant, using lipofectamine 2000 and according to manufacturer’s instructions. 48 h after transfection samples were collected for experiments. Filtrin construct expression was similar in all transfected cells as assessed by filtrin mRNA and protein expression levels.

When indicated, transfected cells were incubated for 14 h with 10 μg/ml of tunicamycin (Sigma, St. Louis, MO).

### Immunoblotting for the ER-Stress proteins

Forty-eight hours post transfection cellular lysates or cellular medium were collected for experiments. Cells were lysed in equal volumes of pre-heated 2xSDS loading buffer (Sodium Dodecyl Sulphate 125 mM, Tris-HCl pH 6.8, 20% Glycerol, 2% SDS, 2% β-mercaptoethanol and bromophenol blue) and homogenized via sonication. Antibodies used against the ER-Stress proteins were: anti-BiP, anti-PERK, anti-P-PERK (Cell Signaling Technology, Danvers, MA) and anti-CHOP, anti-p-eIF2a (SantaCruz Biotechnology, CA), followed by peroxidase-labelled secondary antibodies either goat anti-mouse or donkey anti-rabbit (SantaCruz Biotechnology, CA). Proteins were detected using the Enhanced ChemiLuminescence (ECL) Plus Blotting Detection system (Amersham Biosciences, Buckinghamshire, UK) and were visualized by autoradiography on photographic film (KODAK X-OMAT, NY). All transblots were reprobed with anti-β-tubulin antibody (SantaCruz Biotechnology, CA) to prove equal amounts of protein were loaded on the membrane. Band density was defined using the ImageJ Software (http://imagej.nih.gov/ij).

## Results

### Candidate SNPs genotyping—The emergence of *NEPH3*-p.V353M variant in the HEMATURIA cohort

We used the Polyphen, SNPs3D and SIFT algorithms to predict the likely effect of all registered non-synonymous single nucleotide polymorphisms (SNPs) in 10 genes known to be the most important for the SD structure and function: *NPHS1*, *NPHS2*, *NEPH1*, *NEPH2*, *NEPH3*, *CD2AP*, *TRPC6*, *TJP1 (*known as *ZO1)*, *FAT1*, *FAT2* (see methods for details). SD is considered as one of the most substantial functional parts of the glomerular “sieve”, where its main scaffold is considered to be constructed by the Ig-like domains of the four known nephrin-like proteins (see methods for details about rationale of gene selection). Two SNPs in two genes were predicted to have functional effects on their proteins: *FAT2*-p.G1515S (rs2278370) and *NEPH3*-p.V353M (rs35423326). The two non-synonymous SNPs were initially genotyped in sub-cohort A of the HEMATURIA cohort (Table 1). *NEPH3-*p.V353M (rs35423326) was suggestive for association with severe renal disease (p = 0.036 for genotypic association, Table B in S1 File).

We next genotyped *NEPH3-*p.V353M in three additional hematuric sub-cohorts of the HEMATURIA cohort. Among 301 severely affected patients there were 24 heterozygous and one homozygous genotypes while among 223 mildly affected patients there were three heterozygous genotypes (Table 2). Genotypic association analysis gave a statistical significance of p = 3.0x10^-4^ (OR = 6.64, CI 1.98–22.29) and after adjustment for gender and age gave a p = 3.0x10^-3^. Allelic association analysis gave a statistical significance of p = 2.0x10^-4^ and p = 4.2x10^-3^ after adjustment for patients’ kinships (Table 2). 353M allele frequency in the general population is 2.7% which is similar to that of other populations (www.ensembl.org). Interestingly, the only one homozygous 353M/M patient that was identified, is a 46-year-old man, severely affected with IgA nephropathy, and a GFR of 23 mL/min, not having reached ESRD yet. The results were consistent with Hardy-Weinberg equilibrium in these cohorts.

### Testing for association of *NEPH3*-V353M with microalbuminuria in cohorts of the general population

**Fig 1:**
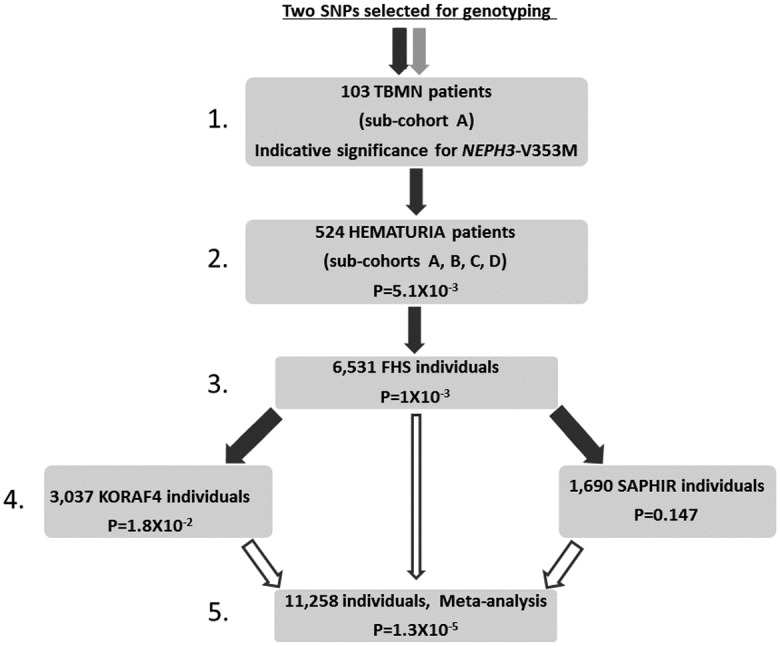
Flowchart of the genotyping strategy followed to investigate the significance of the two SNPs in four genes that emerged to have functional significance based on the *in silico* assay (see material and methods). Black arrow symbolizes the *NEPH3*-V353M variant which initially derived indicative significant association and was investigated further (black not filled arrows symbolize the meta-analysis). Light grey arrows symbolize the three SNPs found not to be significantly associated in sub-cohort A and not tested further. Dot-lined arrows symbolize the two SNPs that were found to be non-polymorphic in this cohort and not tested further.

Genotyping 6,531 subjects of the Framingham Heart Study (FHS) for the *NEPH3*-p.V353M variant revealed association of the rare homozygous genotype 353M/M with microalbuminuria (p = 1.0x10^-3^ adjusting for gender and age, OR = 5.92). No association was found with serum creatinine or cystatin values (results not shown). In order to replicate this result we genotyped the general population cohorts KORAF4 and SAPHIR (Table 3). Statistical analysis confirmed the association with microalbuminuria which, however, did not reach statistical significance in the SAPHIR, perhaps due to the fact that only two homozygous M/M individuals were found (Table 4). Estimates pointed in all three populations in the same direction and a final meta-analysis of all three cohorts, including 11,258 subjects, revealed a p-value of p = 1.3x10^-5^ for the recessive model with an OR of 7.46 (95% CI 2.50–22.23) (Fig 1, Table 4).

**Table 4:** Frequencies and statistics of *NEPH3*-p.V353M in three general population cohorts including a meta-analysis.

		Genotype counts			Genotype frequency			Allele counts		Allele frequency		Statistics		
Cohort	N	VV	VM	MM	VV	VM	MM	V	M	V	M	Dominant model^a^	Recessivemodel^a^	Odds Ratio (Recessive model)
**Controls (without MA)**														
FHS	5,928	5,508	415	5	0.929	0.070	0.001	11,431	425	0.964	0.036			
KORAF4	2,640	2,487	152	1	0.942	0.057	0.001	5,126	154	0.971	0.029			
SAPHIR	1,525	1,423	101	1	0.933	0.066	0.001	2,947	103	0.966	0.034			
**Total (Meta-analysis)**	**10,093**	**9,418**	**668**	**7**	0.933	0.066	0.001	**19,504**	**682**	0.966	0.034			
**Cases (with MA)**														
FHS	603	561	39	3	0.930	0.065	0.005	1,161	45	0.963	0.037	0.946	**1.0x10**^**-3**^	**5.92** (1.41, 24.85)
KORAF4	397	380	15	2	0.957	0.037	0.006	775	19	0.976	0.024	ND	**1.8x10**^**-2**^	**13.36** (1.21, 147.71)
SAPHIR	165	153	11	1	0.927	0.067	0.006	317	13	0.961	0.039	0.738	0.147	**9.29** (0.58, 149.27)
**Total (Meta-analysis)**	**1,165**	**1,094**	**65**	**6**	0.939	0.056	0.005	**2,253**	**77**	0.967	0.033	ND	**1.3x10**^**-5**^	**7.46** (2.50, 22.23)

MA: Micro-albuminuria, as defined in Methods section; ND: Not done

^a^ Adjusted p-values for gender and age

No other *NEPH3* non-synonymous SNPs were found to be linked with the 353M allele, after direct re-sequencing of all the 15 exons of the gene in 12 patients of HEMATURIA cohort. Synonymous variants p.G164G and p.A351A in *NEPH3* were found in partial linkage disequilibrium with V353M, without reaching a statistical significance as modifiers (results not shown).

### Bioinformatic analysis

**Fig 2:**
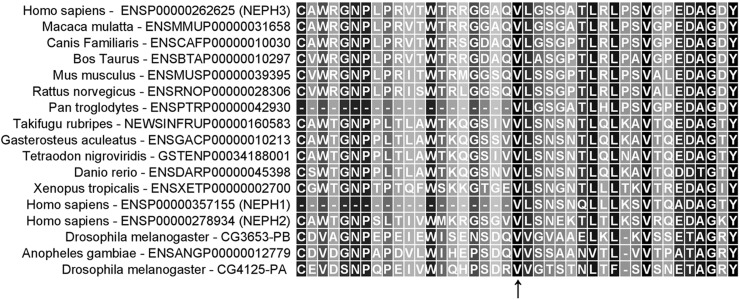
Alignment of orthologs and paralogs sequences of the Neph3 protein (filtrin) around the 353V residue position. Note that there is absolute conservation of the relevant amino-acid residue, across a very broad evolutionary range.

At protein level, valine at position 353 of the protein is highly conserved across evolution (Fig 2) and is located within the 4^th^ Ig-like domain (Neph3 has five Ig-like domains) of the extra-cellular N-terminal region of Neph3 protein. Its substitution by a methionine residue is predicted to abolish a local β-strand domain of the protein (Figure A in S1 File). At the mRNA level, guanine to adenine substitution does not seem to be related with any significant structural change, as tested with the 2D mRNA prediction model using the CLC Main Workbench 6 software package (Figure B in S1 File).

### Assays for Neph3 homo-dimerization and for hetero-dimerization with nephrin

**Fig 3:**
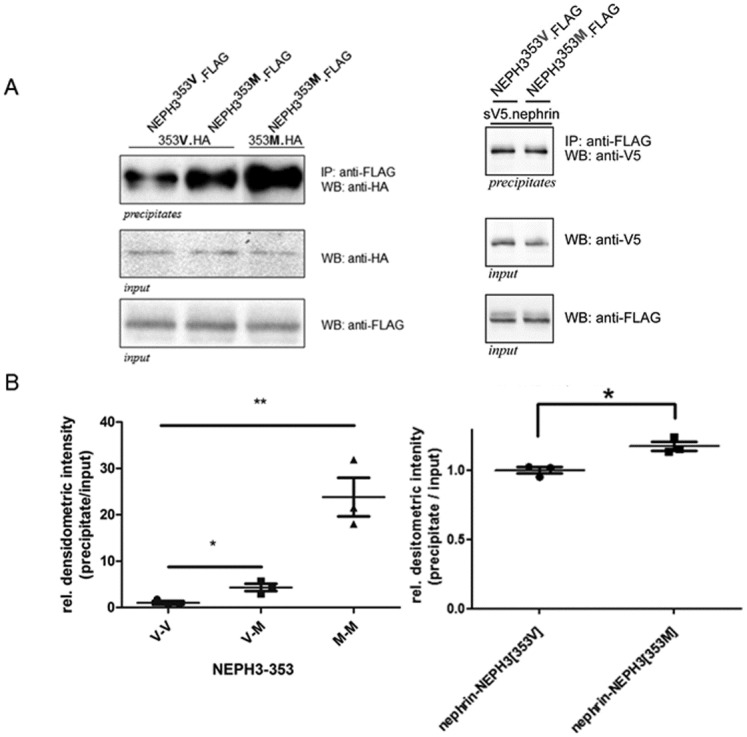
Co-immunoprecipitation experiments, testing for the binding effectiveness of Neph3 protein with methionine (M) at the 353 position. **(A)** Left panel: FLAG-Neph3[353**V**] and FLAG-Neph3[353**M**] were immuno-precipitated with anti-FLAG antibody and then they were analyzed by western blot using an anti-HA antibody (for HA-Neph3[353**V**] and HA-Neph3[353**M**]) in order to check all possible hetero- and homo-dimer interactions. Neph3[353**M**]-Neph3[353**M**] homodimers are strongly increased compared with the Neph3[353**V**]-Neph3[353**M**] and the Neph3[353**V**]-Neph3[353**V**] ones. Right panel: FLAG-Neph3[353**V**] and FLAG-Neph3[353**M**] were immuno-precipitated with anti-FLAG antibody and then they were analyzed by western blot using an anti-V5 antibody (for sV5-Nephrin]. Nephrin-Neph3[353**M**] heterodimers are slightly increased compared with the Nephrin -Neph3[353**V**] ones. Anti-V5 and anti-FLAG western blots from lysates (input) were used for loading normalization. V5-NPHP1 (nephrocystin) served as an experiment control. **(B)** Statistics of densitometry of the blots in Fig 3A, n = 3. Intensity (±SEM) is given as a percentage of wild-type intensity, which is set by definition at 1.0 (100%). There is statistical significance (unpaired t-test) for the homodimerization and heterodimerization comparisons described in Fig 3A. For more details see text.

Co-immunoprecipitation experiments were performed in order to identify any dimerization alterations of the Neph3 protein, in the presence of the 353M variant. Each co-immunoprecipitation experiment was performed in triplicate. Western blot analysis from transiently transfected HEK293T cells revealed strong increase in the homodimer interaction 353M-Neph3/353M-Neph3 variants (mean normalized densitometry value: 23.80 ± 4.166) as compared to the interactions of 353V-Neph3/353M-Neph3 and 353V-Neph3/353V-Neph3 (mean normalized densitometry values: 4.327 ± 0.7765 and 1.000 ± 0.3716 respectively) (Fig 3A). Unpaired t-test was statistically significant with p = 0.0055. Similarly, there was a slight increase in the interaction between the 353M-Neph3 and Nphs1 (mean densitometry value: 1.177 ± 0.033) as compared to the interaction of 353V-Neph3 with Nphs1 (mean normalized densitometry value: 1.000 ± 0.024) (Fig 3B). Unpaired t-test was statistically significant with p = 0.012.

### Overexpressed *NEPH3*-353M variant results in up-regulation of unfolded protein response markers in the presence of tunicamycin

**Fig 4:**
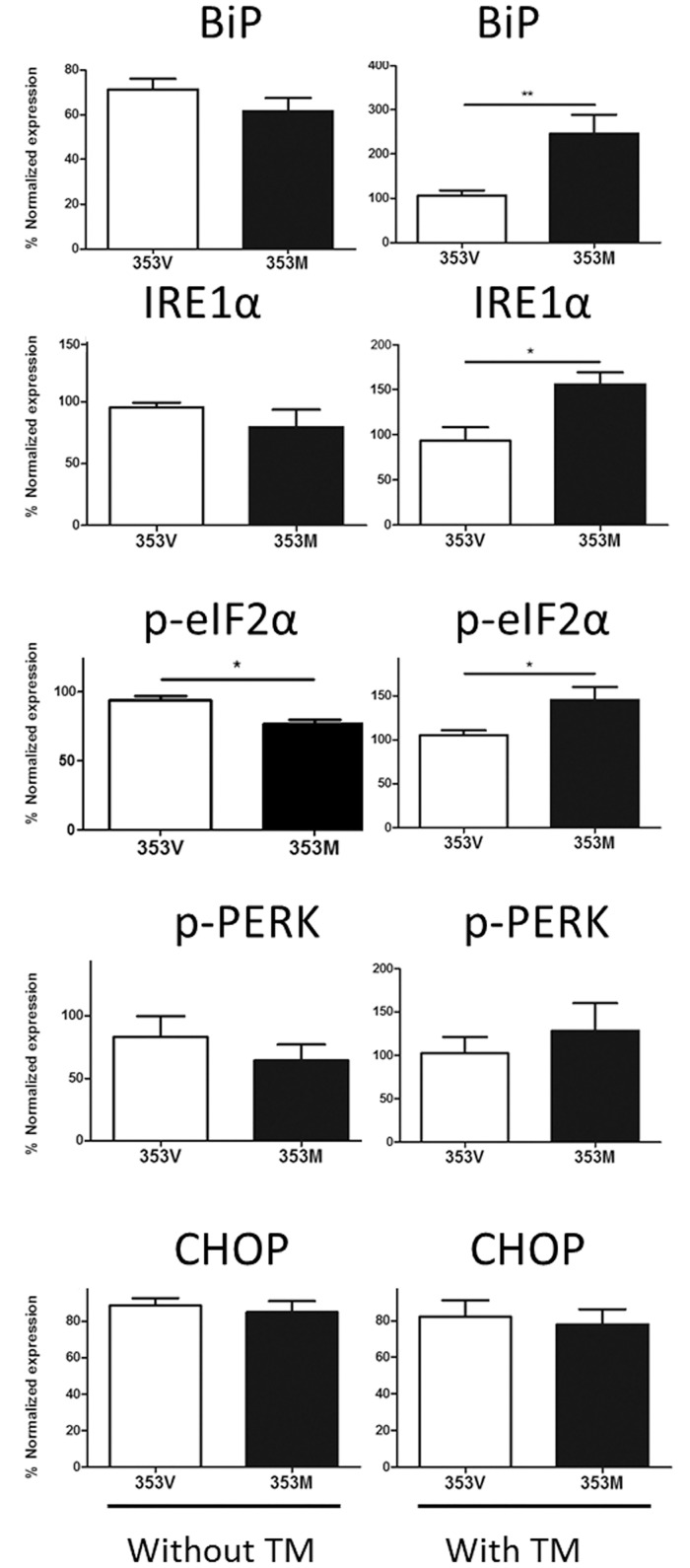
Results of the densitometry analysis of western blots where we assayed the elevation of markers of the unfolded protein response pathway, in the presence of the two *NEPH3* alleles (normal “V” vs mutant “M”). Note that in the presence of tunicamycin (TM, a potent factor adding additional cellular stress), three markers (BiP, IRE1a, p-elF2a) are rising significantly (asterisks mark the significance) for the “M” allele.

In human podocyte AB8/13 cells overexpressing either the 353V or the 353M variant we observed no significant effect in unfolded protein response (UPR) marker elevation. Considering that the 353M variant does not have a pathogenic effect on its own but it is hypothesised to act as a modifier gene on the background of another mutation, it was not particularly surprising that no perceptible differences were observed between the variant and the wild type overexpressing cells. To intensify the stress and to test the susceptibility of the mutant variant expressing cells to other external stress factors, transfected cells were exposed to a potent ER-stressor, as previously described[57]. To this end, transfected cells were treated with 10 μg/ml of tunicamycin, an inhibitor of N-linked glycosylation (Fig 4). Notably, after exposure to tunicamycin, three of the five UPR markers tested (BiP, IRE1a, p-elF2a) were considerably more pronounced in the 353M-expressing cells compared to 353V-expressing cells (Fig 4). P-PERK did not reach statistical significance despite the obvious trend observed in cells transfected with the mutant 353M allele. Only CHOP marker did not show any detectable alteration in expression, in the presence of tunicamycin. These results indicate that the 353M mutant variant is more prone to additional elevation of ER stress, compared to the wild type upon exposure to an external stressor.

## Discussion

Progression in primary hematuric glomerulopathies, inherited or not, is still an open question in nephrology. The phenotypic heterogeneity observed among patients with inherited monogenic disorders, including AS, TBMN and CFHR5 nephropathy, prompted us to hypothesize that the full spectrum of the phenotype behaves as a multifactorial condition, implicating primary genes, modifier genes and environmental factors [34, 37]. Here we considered that excellent candidates to act as genetic modifiers could be non-synonymous SNPs located in specific genes of the SD. Many of these genes have been linked with inherited nephrotic syndromes and corresponding encoded proteins are the main scaffold of the glomerular filtration barrier in the glomeruli. Under these assumptions we hypothesized that specific missense variants on these genes, perhaps acting as hypomorphic mutations, may contribute to progression in a subset of hematuric patients. Following a strategy that is detailed in Methods and Results sections, we focused on filtrin (Neph3), largely of unknown function, which has been shown to interact with various slit diaphragm proteins. After deriving a suggestive significance in a previously well-studied TBMN sub-cohort of 103 patients, we further evaluated this SNP in the pooled hematuric cohort, HEMATURIA, with 524 patients (Table 1), comprised mainly by TBMN and IgA nephropathy patients. Statistical analysis revealed a high risk for the carriers of this variant (genotypic association: p = 3.0x10^-3^, OR = 6.63 adjusting for gender/age; allelic association: p = 2.0x10^-4^) (Table 2). Pending replication by other researchers, our study suggests that variant *NEPH3*-V353M may have a prognostic value for an adverse outcome when it occurs in patients with a background of another primary hematuric glomerulopathy, such as TBMN or IgA nephropathy. This makes the variant qualify as a hypomorphic allele which on its own is not adequate to cause any perceptible symptom, when in heterozygosity. A similar inheritance pattern explained some rare severe and early onset cases of autosomal dominant polycystic kidney disease when hypomorphic *PKD1* mutations were co-inherited with variants in the *PKHD1* or the *HNF-1β* gene [58, 59].

**Fig 5:**
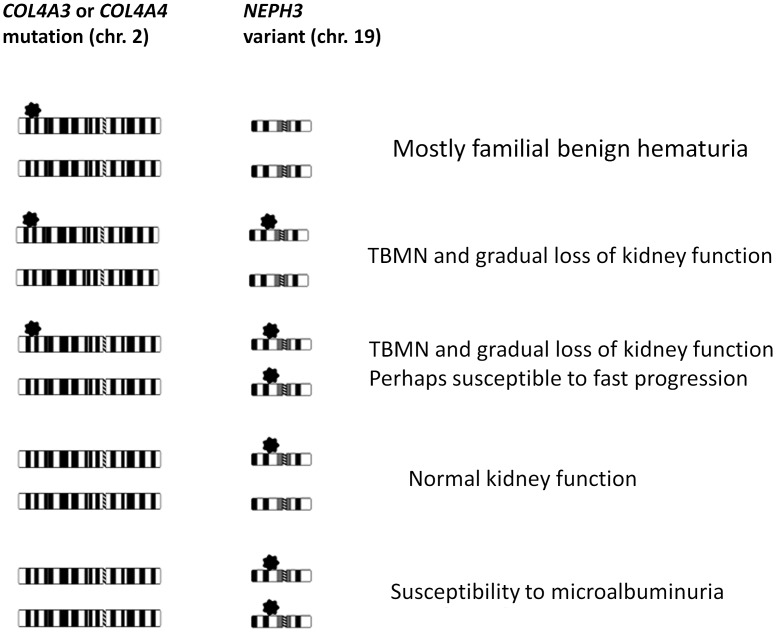
A proposed hypothetical model for the mechanism of disease development when a causative heterozygous mutation is inherited on the *COL4A3/A4* gene or when a heterozygous mutation is co-inherited with a variant on a genetic modifier. This general model implies that hypomorphic mutations such as *NEPH3*-p.V353M are benign in heterozygosity on their own but may confer malfunction and impair the integrity of the glomerular filtration barrier when co-inherited with a collagen IV pathogenic mutation, or in homozygosity.

We next asked whether this variant is associated with evidence of kidney disease in the general population. A search in ExAC genome browser revealed that the M allele frequency is 0.0305 in 54,373 healthy genomes, which is lower than the frequency found in the Cypriot general population, of 0.054 (Table 2), therefore it is not a truly rare variant [60]. Genotyping and analyzing 6,531 subjects of the FHS revealed association of the homozygous genotype 353M/M with microalbuminuria in this population (p = 1.0x10^-3^ adjusting for gender and age, OR = 5.92) (Table 4). In order to confirm this result we further genotyped *NEPH3*-p.V353M in the two general population-based samples SAPHIR and KORAF4. A meta-analysis for all three cohorts, demonstrated that 353M/M homozygotes inherit a high risk for microalbuminuria (adjusted p = 1.3x10^-5^, OR = 7.46). These data are consistent with the hypothesis that one dose of this hypomorphic mutation is sufficient to predispose to kidney impairment on the background of a primary hematuric glomerulopathy whereas two doses are needed to predispose an otherwise healthy individual to manifest microalbuminuria, a sign suggesting glomerular filtration barrier instability (see Fig 5 for our suggested model). We would like to underline that the results suggest that microalbuminuria is not always of endothelial origin[61], as it is believed today, but it can be of podocytes’ origin. Interestingly, a recent study gave additional evidence for the role of filtrin in prevention of the glomerular protein leakage, since it was shown that non-synonymous variants in *NEPH3* gene can cause nephrotic syndrome in dogs[62].

p.V353M is located in the 4^th^ Ig-like domain of filtrin. Ig-like domains are regions of interactions between members of the nephrin family of proteins. 2D Neph3 structure prediction showed a possible alteration caused by the methionine substitution (Figure A in S1 File). The co-immunoprecipitation experiments show that this change from valine to methionine at position 353, drives to a much stronger homodimerization of Neph3-353M/Neph3-353M compared to Neph3-353M/Neph3-353V and Neph3-353V/Neph3-353V. Additionally, Neph3-353M/Neph3-353V interaction seems to be also stronger than Neph3-353V/Neph3-353V. Importantly, the results show the same trend for Neph3 –Nephrin heterodimerization, as valine to methionine at 353 increases the Neph3 –Nephrin interaction (Fig 3). We propose that a functionally deleterious variant, like 353M, can facilitate the degeneration of the SD integrity by inducing alterations of different protein interactions, during the long aging process in a healthy individual, or most significantly on the background of another primary glomerular disease.

UPR is a sensitive cellular procedure taking place in the ER where unfolded proteins (e.g. due to a mutation) are perceived by the cell and consequently activating a cascade of events aimed at restoring ER homeostasis. This mechanism proved to be significant in kidney cells [63, 64]. We wish to point out that in recent work we showed that collagen IV mutations which cause Alport syndrome and/or TBMN, activate the UPR signalling cascade in cultured podocytes, in human biopsies and in a mouse knockin model[35, 65]. In the present work, our investigation for potential activation of the ER stress-dependent signalling, included examination of the protein levels of the UPR markers BiP, IRE1a, p-eIF2α, p-PERK, and CHOP in cells overexpressing the 353V or the 353M variant of filtrin. UPR marker activation was similar both for the 353V and the 353M overexpressing cells. In order to simulate a more realistic environment that the podocyte has to anticipate in the glomerulus, stress was enhanced by exposure of transfected cells to tunicamycin, a pharmacological ER stress activator. In this setup the stress pressure imposed on the cells was doubled: 1) genetic background of the filtrin and 2) exposure to the stress-causing agent. Interestingly, the cells expressing the 353M variant were prone to further elevating UPR markers than the WT expressing cells (Fig 4). This is interesting since one might hypothesise that the 353M variant could potentially raise susceptibility of the cell in the presence of other predisposing factors, genetic or environmental, such as an inherited pathogenic mutation (like in TBMN patients), a locally increased osmotic or mechanical pressure, toxic chemicals etc. We conclude that variant *NEPH3*-p.V353M imposes a slowly degenerating process on podocytes that needs several decades to manifest.

The candidate gene approach we followed here, where we filtered all non-synonymous variants in ten SD genes using a bioinformatic approach, may be ideal for diseases like glomerulopathies where there is plenty of information for the proteins taking part at the associated biological micro-structures (*e*.*g*. the SD), especially when dealing with rare variants. Even though GWAS are attractive and popular, the number of patients in our cohorts would not support them. From a practical perspective, in our cohorts of patients with microscopic hematuria, the results imply that testing for the presence of the *NEPH3-*p.V353M, has a highly positive predictive value for renal impairment. This, however, needs to be evaluated further in appropriate prospective cohort studies.

## Conclusion

We provided three lines of evidence based on evolutionary conservation, genetic association studies and functional assays, which support the role of variant *NEPH3*-p.V353M as a hypomorphic mutant with low or incomplete penetrance. Our findings may afford the opportunity for early detection of a subgroup of patients with glomerular hematuria who are at increased long-term risk of kidney function decline. With this in mind, this finding conforms to the low-frequency variant-large effect hypothesis in opposition to the common variant hypothesis. At the same time, homozygosity in the general population may also arise as a risk factor for micro-albuminuria of unknown significance to health. Admittedly, it is more than obvious that more genetic variants (perhaps hundreds or thousands), and environmental factors exist that contribute to this end-phenotype, and waiting to be identified. In our view, every family or patient could have their own additional variant that either predispose them to, or protect them from, adverse renal function developments, on the background of another primary glomerulopathy. At the same time, these same variants may predispose subjects of the general population, when inherited in homozygosity. If this is the case, molecular examinations of prognostic value could be massive parallel sequencing of panels of candidate genes, something that nowadays has become easier and cheaper with Next Generation Sequencing technologies.

## Supporting information

S1 File Further bioinformatic analysis of filtrin protein and filtrin mRNA, genotypic information, description of KORA F4 and SAPHIR studies. (DOC)
