# Supplementary material for: A functional variant in NEPH3 gene confers high risk of renal failure in primary hematuric glomerulopathies. Evidence for predisposition to microalbuminuria in the general population
Source: PLoS One. 2017 Mar 23;12(3):e0174274. doi: 10.1371/journal.pone.0174274 (PMC5363870; doi:10.1371/journal.pone.0174274)
Supplement: S1 File — (DOC) [file pone.0174274.s001.doc]

**SUPPORTING INFORMATION**

**Figure A**


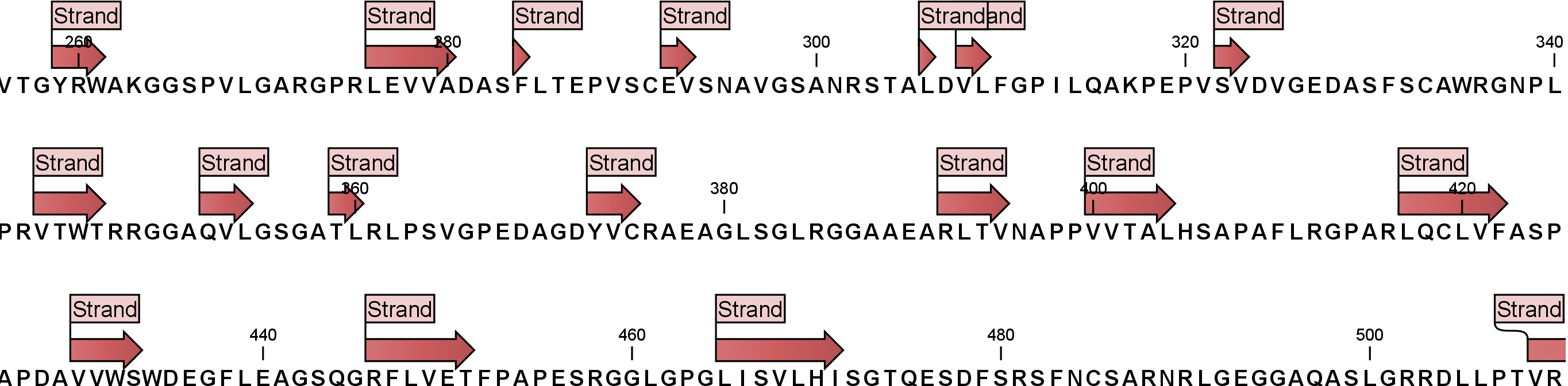

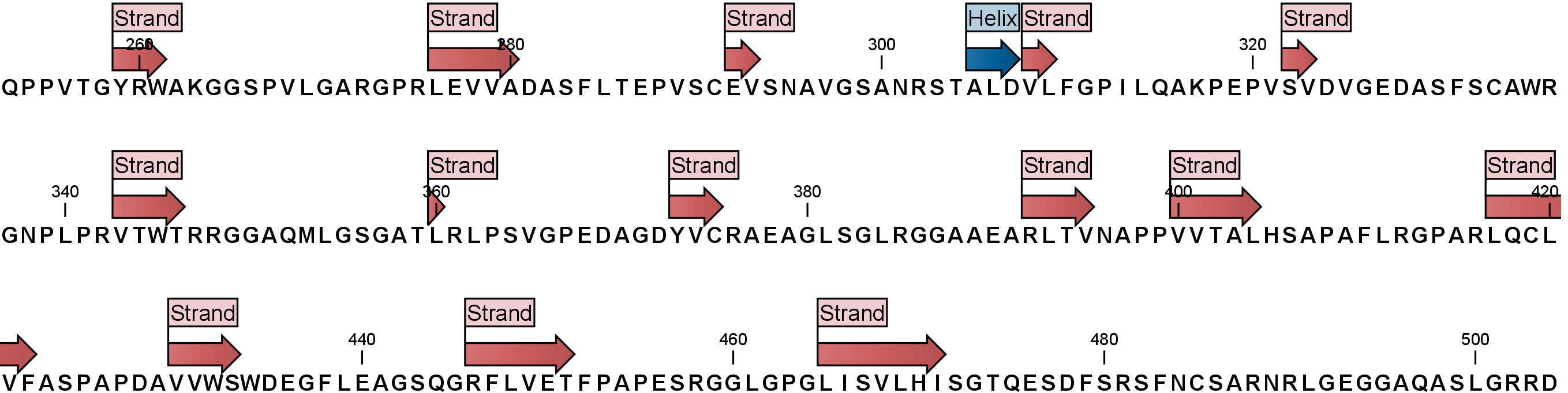


**2D protein structure prediction of the two variants of Neph3 (filtrin) protein (353V, 353M).** In red box is the 353 amino-acid residue and neighboring residues. It is predicted that methionine substitution causes a conformation change due to loss of a β strand.

**Figure B**


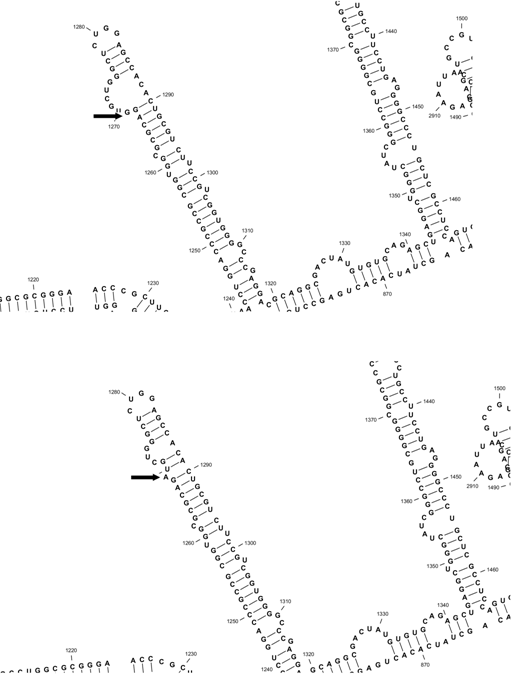


**Minimum free energy of structure: ΔG = -1301,6 kcal/mol**

**Minimum free energy of structure: ΔG = -1301,1 kcal/mol**

**2D mRNA structure prediction of the two variants of *NEPH3* mRNA (GTG: Val, ATG: Met; black arrows).** Minimum free energy estimation is similar for both variants, indicating that no significant changes occur in mRNA secondary structure.

**Table A.** **Genotype associations for two SNPs in sub-cohort A.** P-values were calculated by Fisher’s Exact Test (2-sited). Homozygous patients for the (22) genotypes were not identified.

| SNP | Alleles | Genotypic counts | | (22)+(12) vs (11) | |
| --- | --- | --- | --- | --- | --- |
|  | 1 2 | Mild | Severe | p-value | OR (95% CI) |
| ***NEPH3-*V353M** (rs35423326) | **V M** | VV (44) | VV (53) | **0.036** | **NA** |
|  |  | VM (0) | VM (6) |  |  |
| *FAT2*–G1515S (rs2278370) | G S | GG (40) | GG (51) | 0.550 | 1.57 (0.44, 5.58) |
|  |  | GS (4) | GS (8) |  |  |

NA: Not applicable, as Odds Ratio cannot be estimated due to zero genotypic values in the “Mild” category.

OR: Odds Ratio; CI: Confidence Intervals

**Table B.** **PCR primers and restriction enzymes used for SNPs genotyping in this study.**

| SNP | Forward primer | Reverse primer | PCR size (bp) | Tm (^0^C) | Restriction enzyme | Cleavage products (bp) |
| --- | --- | --- | --- | --- | --- | --- |
| *NEPH3*–V353M | CTAGAGGGTGTGGTGTTTCTGTG | CTAAAAGCCTCGCCTTCAATAG | 590 | 60.5 | *EcoNI* | 300 + 180 + 82 + 28 (V allele) |
| *FAT2*–G1515S | CAACTCTGTCTCGTGCATGG | CAGCTGGACCCAAGCAGT | 209 | 58 | *AluI* | 156 + 53 (S allele) |

**Text A. Analytical description of KORAF4 and SAPHIR cohorts**

**KORA F4 Study:** The KORA surveys for genetic research have been described in detail previously[^51-52^](#_ENREF_51) and have been initiated as part of the MONICA (Monitoring of Trends of Cardiovascular Diseases) multi-center study. The fourth third KORA survey (KORA S4) is a population-based sample from the general population of the South-German city of Augsburgand surrounding counties, recruited 1999-2001. All participants had a German passport and were of European origin. Using stored urine samples, urinary albumin concentration was measured with a latex enhanced nephelometric assay (Siemens Healthcare Diagnostics) on a Dade Behring BN2 apparatus. Urinary creatinine concentration was measured using a kinetic Jaffe method.

**SAPHIR Study**: The "Salzburg Atherosclerosis Prevention Program in subjects at High Individual Risk"(SAPHIR) is an observational study conducted in the years 1999-2002 involving healthy unrelated subjects. In the study at hand, we had genotypes available from 1720 individuals: 638females from 39 to 67 years of age and 1082 males from 39 to 66 years of age. Study participants were recruited by health screening programs in large companies in and around the city of Salzburg as described recently[^53^](#_ENREF_51). All individuals were of West-Eurasianorigin. Subjects with established coronary artery, cerebrovascular or peripheral arterial disease, congestive heart failure, valvular heart disease, chronic alcohol (more than three drinks a day) or drug abuse, severe obesity (BMI>40kg/m²) and pregnant women were excluded. Informed consent was obtained from each participant. At baseline all study participants were subjected to a comprehensive screening examination. A detailed personal and family history was assessed via standardized questionnaires. A physical examination included measurement of anthropometric parameters such as weight, height, waist circumference and percentage body fat. Blood samples were collected after an overnight fasting period. Urinary creatinine (mg/dl) was measured using a modified kinetic Jaffe reaction (CREA®, Roche Diagnostics GmbH, Mannheim, Germany); Urinary albumin concentration (mg/l) was determined using the Tinaquant® assay (Roche Diagnostics GmbH, Mannheim, Germany).
